# The antiviral activity of myricetin against pseudorabies virus through regulation of the type I interferon signaling pathway

**DOI:** 10.1128/jvi.01567-24

**Published:** 2024-11-27

**Authors:** Yizhen Song, Xufan Zhao, Yaqin Chen, Xingyue Yu, Tianli Su, Juan Wang, Tingke He, Zhongqiong Yin, Renyong Jia, Xinhong Zhao, Xun Zhou, Lixia Li, Yuanfeng Zou, Mingyue Li, Dongmei Zhang, Yingying Zhang, Xu Song

**Affiliations:** 1 Natural Medicine Research Center, College of Veterinary Medicine, Sichuan Agricultural University 506176 , Chengdu, China; 2 College of Animal Science and Technology, Sichuan Agricultural University 506176 , Chengdu, China; Lerner Research Institute, Cleveland Clinic, Cleveland, Ohio, USA

**Keywords:** antiviral activity, flavonoids, pseudorabies virus, type I interferon signaling pathway

## Abstract

The type I interferon signaling pathway constitutes a pivotal component of the innate immune response, encompassing the cGAS/STING and JAK/STAT pathways. Drugs that affect the body’s innate immune response could potentially be used as broad-spectrum antivirals. In this study, the antiviral activities of 25 flavonoids against pseudorabies virus (PRV) were tested in PK-15 cells. Eight active flavonoids were identified, with IC_50_ values ranging from 23.24 to 323.09 µM. Subsequently, the regulatory effects of these flavonoids on the cGAS/STING pathway in PRV-infected cells were investigated. It was found that Myricetin significantly increased the transcriptional levels of *cGAS*, *STING*, *IRF3*, and *IFN-β*, which had been reduced by PRV infection. The regulation of the type I in

terferon signaling pathways by myricetin following PRV infection was further investigated through the production of cGAMP and the assessment of transcriptional and protein levels of pivotal genes and proteins. To confirm the activation of the innate immune response, a dual luciferase gene reporter study found that the expression of the IFN-β promoter in the myricetin-treated group was significantly elevated in a cellular model of type I interferon signaling pathway, and the contents of IFN-β were also significantly higher than those observed in the infected-untreated group in a PRV-infected mice model. Moreover, the transcriptional and protein levels of key genes and proteins in cell and mouse models exhibited analogous outcomes to those observed in PRV-infected cells. These findings suggest that myricetin can effectively activate the type I interferon signaling pathway, thereby enhancing the innate immune response during PRV infection.

**IMPORTANCE:**

PRV, belonging to the *Herpesviridae* family, is an easily overlooked zoonotic pathogen that can threaten human health. The immunoprotective efficacy of conventional vaccines is significantly reduced due to the continuous mutation of the PRV genome, which constantly generates new viral strains. Therefore, there is a need to develop potent therapeutic drugs. PRV is capable of evading the host's natural immunity by suppressing the host's type I interferon signaling pathway, and the search for drugs that activate natural immunity can induce the body to produce type I IFN interferon and exert antiviral effects. Accordingly, the present study sought to identify active compounds from flavonoids that modulate the type I IFN interferon signaling pathway and thus inhibit the proliferation of PRV, which provides a new idea for the development of anti-PRV drugs from flavonoids that modulate the type I IFN interferon signaling pathway to enhance the body's antiviral immunity.

## INTRODUCTION

Pseudorabies virus (PRV), also known as porcine herpesvirus type 1, is specifically classified within the subfamily of α-herpesviruses in the genus *Varicella* virus (1, 2). Herpesviruses exhibit a broad spectrum of transmission, with the potential to induce a multitude of severe illnesses, particularly affecting the central nervous system (3). Moreover, it has the capacity to establish a latent infection in the infected individual (4). As an easily overlooked zoonotic pathogen within this family, there has been a notable increase in the number of case reports on human infections with PRV in recent years (5). Since 2017, China has reported 25 cases of human infection with PRV, indicating that PRV not only threatens the health of animals but also poses a potential threat to humans (6).

Upon entering cells, dsDNA fragments of herpesviruses can activate the host innate immune response through the cGAS-STING-TBK1-IRF3 recognition pathway (7, 8). This, in turn, induces interferon production and further promotes the generation of interferon-stimulated genes (ISGs) through the JAK/STAT pathway (9, 10). Herpesviruses employ a multitude of strategies to achieve the inhibition of the cGAS/STING signaling pathway, including the masking of double-stranded DNA presentation, the modulation of the activation state of cGAS and STING proteins, and the degradation of cGAMP, which collectively facilitate immune evasion (10). The ICP0 protein induces the degradation of p50 in host cells through its ubiquitin ligase activity and directly inhibits the phosphorylation of p65 into the nucleus, thereby inhibiting the type I interferon response (8). The UL36 protein deubiquitinates the TNF receptor-associated factor 3 (TRAF3) through the activity of its deubiquitinating enzyme structural domain USP, thereby blocking the response of this factor to TBK1 (11,12); this ultimately inhibits the synthesis of IFN-I. The US3 protein, when released into host cells, blocks the normal phosphorylation of transcription factors IRF3 and p65 into the nucleus and serves to inhibit the transcriptional expression of IFN-β (13, 14). Furthermore, the US3 protein has an inhibitory effect on the expression of intracellular cytoplasmic TLR3 proteins, thus limiting the occurrence of the corresponding immune response (15). The UL41 protein has been demonstrated to directly inhibit the mRNA expression of ISGs, including Viperin, ZAP, IFIT3, and Ch25h, which may facilitate immune evasion (16–19).

Flavonoids have been demonstrated to affect specific steps in the life cycle of a range of viruses, including herpesviruses, influenza virus, hepatitis virus, dengue virus, and Zika virus (20–22). Myricetin and kaempferol have been demonstrated to inhibit the replication of herpes simplex virus (HSV) and PRV via the EGFR/PI3K/Akt pathways (23, 24). Baicalein has been demonstrated to play an inhibitory role in the replication of HSV-1 and HSV-2 by modulating various cell signaling pathways, including PI3K-Akt, p53, NF-κB, and MAPK pathways (25). Naringenin is more potent in inhibiting viral particle assembly of hepatitis C virus, whereas quercetin inhibits viral translation by blocking non-structural protein 5A (NS5A) and internal ribosomal entry site (IRES)-mediated viral translation, as well as heat-shock protein 70 (HSP70)-induced inhibition of viral translation (26, 27).

In this study, we first tested the antiviral activities of 25 flavonoids against PRV, and then, the active compounds were further evaluated for the regulation effects on the cGAS/STING and JAK/STAT signaling pathways. This provides a novel insight into the development of anti-herpesvirus drugs from flavonoids by regulating the type I interferon signaling pathway.

## MATERIALS AND METHODS

### Cell, virus, and plasmids

Porcine kidney cells (PK-15) were grown in Eagle’s minimal essential medium (EMEM) supplemented with 10% (vol/vol) bovine calf serum (Gibco), 100 U/mL penicillin, and 100 g/mL streptomycin. For the maintenance medium (MM), serum concentration was reduced to 2%.

Pseudorabies virus (Ra strain) was purchased from the China Veterinary Microbial Strain Collection Center (Beijing, China) and proliferated in PK-15 cells. The 50% tissue culture infectious dose (TCID_50_) was determined as 10^-7.5^/mL. cGAS-HA, STING-flag plasmids were synthesized and constructed by Sangyo Bioengineering Co, Ltd., (Shanghai, China) and incorporated into the pcDNA3.1 vector. The pGL3.0-IFN-β-luc plasmid was purchased from Transworld Kewei Biotechnology Co, Ltd. (Chengdu, China).

### Cytotoxicity assay of flavonoids

The flavonoids were dissolved in DMSO and diluted with cell culture medium (DMSO ≤0.5%), and added into a 96-well plate containing the monolayer cells. Six replicates were set up for each concentration, as well as blank control cells. After incubation for 48 h, the CCK-8 reagent (MA0218, Meilun, Dalian, China) was then added. The absorbance was measured at 450 nm. The half toxicity concentration (CC_50_) of different flavonoids was then calculated according to the Reed-Muench method (28).

### Viral inhibition assay

Virus suspension (100 TCID_50_; 100 µL) was added to the test wells, and then, the plates were incubated at 37°C for 1 h, allowing virus penetration into cells. After washing three times with PBS, the 2-fold diluted flavonoids within the non-toxic concentration range were added. The blank control and infected-untreated control groups were treated with a cell culture medium. Upon reaching 80% cytopathic effect in the infected-untreated control group, the cell medium was aspirated, and the cells were washed with PBS three times, followed by the addition of a CCK-8 reagent (MA0218, Meilun; Dalian, China). The absorbance was measured at 450 nm. The half-inhibitory concentration (IC_50_) of different flavonoids was calculated according to the Reed-Muench method.

### Real-time PCR assay

Different concentrations of flavonoids were added to PRV-infected cells (MOI = 5) in a six-well plate. The uninfected control and infected-untreated control groups were set up. After incubation, cells were then washed by PBS three times, followed by total RNA extraction using the TRIzol agent (RA101-01, Biomed; Beijing, China). This was reverse transcribed to cDNA using the M-MLV 4 First-Strand cDNA Synthesis Kit agent (MT403-01, Biomed; Beijing, China), which was then used to detect the transcriptional levels of test genes in each sample using Hieff UNICON Universal Blue qPCR SYBR Master Mix agent (11184ES08, Yeasen; Shanghai, China), and the primers used are listed in Table 1. The PCR cycling was performed at 95°C for 3 min, followed by 40 cycles of cycling at 95°C for 10 s, 59.8°C for 30 s, and 55°C for 5 s. The Ct value of the target gene was normalized in relation to the expression of the internal reference gene, β-actin. The relative mRNA expression level of each target gene was calculated according to the 2^-ΔΔCt^ method.

**TABLE 1:** Primer sequences used for real-time PCR

Gene	Forward primer sequence (5′ → 3′)	Reverse primer sequence (5′ → 3′)
cGAS (pigs)	TTCTTTCACGTCTGTACCC	GCAGAAAATATGCCACAC
cGAS (mouse)	GTCGGAGTTCAAAGGTGTGGA	GACTCAGCGGATTTCCTCGTG
STING (pigs)	CCGCCTCATTGTCTACCAG	TGCCCATGGTAACCTCC
STING (mouse)	TCGCACGAACTTGGACTACTG	CCAACTGAGGTATATGTCAGCAG
TBK1 (pigs)	CAGCGTGGCTAAGGCAATAA	CATCGTATCCCCTTTCGCAT
TBK1 (mouse)	ACTGGTGATCTCTATGCTGTCA	TTCTGGAAGTCCATACGCATTG
IRF3 (pigs)	AAGGTTGTCCCCATGTGTC	TGTACTGGTCGGAGGTGAG
IRF3 (mouse)	CTGACAATAGCAAGGACCCTTA	AGGCCATCAAATAACTTCGGTA
IRF7 (mouse)	TGAGCGAAGAGAGCGAAGAG	CCAGTAGATCCAAGCTCCCG
IRF9 (pigs)	AGAAGGAGGAGGACGAGGTT	AGACTCAGGGCTGTTGCTG
IFN-β (pigs)	AGTGCATCCTCCAAATCGCT	GCTCATGGAAAGAGCTGTGGT
IFN-β (mouse)	TCCGAGCAGAGATCTTCAGGAA	TGCAACCACCACTCATTCTGAG
JAK1 (pigs)	GTATGGCGGCATTCTCCAAA	TACTGCCCCTGAGCAAAGAG
STAT1 (pigs)	TCTGGCACAGTGGCTAGAAAATC	GAAAACGGATGGTGGCAAAC
ISG54 (pigs)	AAGAACTCCTTGGAGAGCTG	CCTGTATGTTGCACATCGTG
ISG54 (mouse)	CACTGGAGAGCAATCTGCGA	GCCAGTCATCCAGACGGTAG
ISG15 (pigs)	GGGAGTATGACCTCAAGCCTA	ACACTCAATTTTGCCACAGC
ISG56 (pigs)	GACCTACGTCTTCCGACACG	CTTCTGCTTTGCTGTGGTCG
ISG56 (mouse)	TTTACAGCAACCATGGGAGAGA	AGCTTCCATGTGAAGTGACATCT
β-actin (pigs)	GGACTTCGAGCAGGAGATGG	AGGAAGGAGGGCTGGAAGAG
β-actin (mouse)	GGCTGTATTCCCCTCCATCG	CCAGTTGGTAACAATGCCATGT

### cGAMP production assay

The 12-well plate containing a PK-15 monolayer was infected with PRV (5 MOI), followed by treatment with 500 µM myricetin. After incubation for 1 h, the cells were washed with PBS and lysed with 100 µL/well of hypotonic lysate solution on ice for 30 min, after which the lysate was centrifuged at 12,000 rpm for 5 min at 4°C. The resulting supernatant was extracted. Super Nuclease (1,000 U/mL) was added to the supernatant, incubated at 37°C for 30 min, then heated in a 95°C water bath for 5 min, and then centrifuged at 12,000 rpm, 4°C for 5 min, and the supernatant was retained. The supernatant was then mixed with a permeabilization solution in a 1:10 ratio and added to the PK-15 cells, which were then cultured for 30 min. The cells were then switched to a 10% complete medium and cultured for 6 h. Then, the total RNA was extracted from each experimental group, and RT-PCR was performed to detect the transcriptional level of IFN-β in the PK-15 cells.

### Indirect immunofluorescence

PK-15 cells were inoculated into a 6-well plate, and the monolayer was infected with 1 MOI of PRV and then incubated with 500 µM myricetin. After incubation for 12 h, the cells were washed with PBS and fixed with 1 mL of 4% paraformaldehyde (AR1068, Boster; Wuhan, China) per well at room temperature for 1 h. The cells were then washed with PBS three times, before being treated with 0.2% Triton X-100 (T8200, Solarbio; Beijing, China) for 1 h at room temperature. They were then washed again with PBS and treated with 5% bovine serum albumin (BSA) for 5 min. The cells were incubated with p-IRF3 (Bioss, Beijing, 1:4,000) overnight at 4°C. After washing, the cells were incubated with the HRP Conjugated AffiniPure Goat Anti-rabbit/mouse IgG (H + L) (BA105, Boster; Wuhan, China) for 1 h under light protection. After washing, fluorescence microscopy was used to visualize the fluorescence.

### Western blotting assay

Western blotting was employed to examine the protein expressions of cGAS-STING (cGAS, STING, TBK1, IRF3, and IRF7) and JAK-SATA (STAT1, P-STAT1, ISG15, and OAS1) signaling pathways. The PK-15 cells were infected with or without PRV (MOI = 1) for 1 h. The cells were washed three times with PBS and incubated with a culture medium with or without myricetin. The proteins were extracted with the total protein extraction reagent for the cultured cells kit (AR0103-10, BOSTER; Wuhan, China) at 2, 6, and 12 hpi, respectively. The total proteins were subjected to 10% sodium dodecyl sulfate-polyacrylamide gel electrophoresis (SDS-PAGE) for 100 min and subsequently transferred to polyvinylidene difluoride (PVDF) membranes (Millipore, USA). Subsequently, the membranes were blocked with 5% BSA at room temperature for 90 min (Sigma, USA). Primary antibodies directed against IRF3 (1:5000, #11312–1-AP; Proteintech, Wuhan, China), p-IRF3 (1:4000, #29528–1-AP; Bioss, Beijing, China), β-actin (1:5000, #bs-0061R; Boster, Wuhan, China), anti-HA (Abmart, Shanghai,1:5000), anti-flag (Abmart, Shanghai, 1:4000), TBK1(1:4000, #28397–1-AP; Proteintech, Wuhan, China), IRF3 (1:5000, #11312–1-AP; Proteintech, Wuhan, China), STAT1 (1:10000, #66545–1-IG; Proteintech, Wuhan, China), P-STAT1 (1:500, #orb7016; Biorobyt, USA), ISG15 (1:2000,#89771; CST, USA), and OAS1 (1:1000, ab272492; Abcam, USA) were applied at 4°C overnight, respectively. The membranes were washed four times with Tris-buffered saline containing 0.1% Tween 20 (TBST) and then incubated with horseradish peroxidase-conjugated secondary antibody (1:5000, #58802; CST, USA) at room temperature for 1 h. After washing, the proteins were then visualized using enzymatic chemiluminescence (ECL) reagents (BL520A, Bio-sharp; Anhui, China). The expression levels of total proteins were normalized according to the expression of β-actin, and ratios of protein band intensities were obtained using the ImageJ software (Version 1.47; NIH, USA).

### Plasmid cotransfection

Synthetic plasmids cGAS-HA, STING-flag, and IFN-β-luc were subjected to transformation culture, and the plasmid concentrations were screened in accordance with the relevant literature (29). Once the 293T cells had reached 70%–90% confluence in the 6-well plate, the cGAS-HA, STING-flag plasmid, and the empty plasmid were transfected into the cells in accordance with the instructions for the Lipofectamine 3000 transfection reagent (L3000008, Thermo, USA). After transfection for 8 h, the cells were then infected with PRV and treated with myricetin. The western blotting assays were conducted as described above.

### Dual-luciferase reporter gene assay

Once the density of 293 T in the 24-well plate reached 70%–90%, transfection was carried out using the construct plasmids, including the IFN-β-Luc reporter plasmid, cGAS-HA plasmid, STING-flag plasmid, and pRL-TK plasmid. The cells were co-transfected with these plasmids in accordance with the instructions for the Lipofectamine 3000 transfection reagent (L3000008, Thermo, USA), and a control group was set up. Additionally, an IFN-β-Luc empty plasmid was set up at the same time. Following the transfection, the plasmids were replaced with a cell maintenance medium containing virus in the presence or absence of myricetin. After culturing for 12 h, the cells were collected for a Dual-Luciferase reporter gene assay. The Dual-Luciferase Reporter Assay System (E1910, Promega, USA) reagents were employed in accordance with the manufacturer’s instructions.

### Animals, *in vivo* experimental design, and sample collection

Thirty SPF 6-week-old female mice (18 ± 2 g) were pre-fed for 7 days to facilitate their adaptation to the experimental environment. All procedures involving animals and their care in this study were approved (No. 20220102) by the Ethics Committee of Sichuan Agricultural University according to the Regulation of Experimental Animal Management (State Scientific and Technological Commission of the People’s Republic of China, No. 2, 1988) and The Interim Measures of Sichuan Province Experimental Animal Management (Science and Technology Bureau of Sichuan, China, No. 25, 2013).

The mice were randomly assigned to one of three groups (*n* = 10), comprising a mock group, a PRV-infected group, and an infected-treated group (myricetin, 100 mg/kg). The mice in the infected-untreated and myricetin-treated groups were intraperitoneally injected with 0.1 mL of 2 × 10⁴ TCID₅₀ PRV. At 1 hpi, the mice in the myricetin-treated group were administered 0.2 mL of myricetin orally for 5 consecutive days. The mice in the mock and infected-untreated groups were administered an equal volume (0.2 mL) of a 0.5% CMC-Na solution. At 4 dpi, the mortality rate in the infected-untreated group was 40%, whereas in the myricetin-treated group, it was 70%. Subsequently, the surviving mice in each group were euthanized via cervical dislocation. The brain, kidney, heart, liver, lung, and spleen tissues were excised and frozen in liquid nitrogen and then stored at −80°C.

Tissue samples were ground under liquid nitrogen freezing, and then, about 20 mg of tissues was used for RNA extraction using the TRIzol method (RA101-01, Biomed, Beijing, China). The mRNA expression levels were detected as described above. About 20 mg of brain and kidney samples was used for total protein extraction according to the manufacturer’s instructions (AR010, Boster, Wuhan), and then, western blotting assay were performed as described above.

The tissues were weighed (approximately 20 mg) and homogenized using a tissue homogenizer. Subsequently, the homogenate was subjected to centrifugation at 10,000 rpm for 20 min, after which the supernatant was collected to evaluate the content of IFN-β through the use of an ELISA assay. The aforementioned procedure was conducted in accordance with the instructions provided by the kit manufacturer (ml063095, Meilian, Shanghai). The absorbance value of the standard provided in the kit was utilized to establish a corresponding regression curve equation, which was then employed for the calculation of the content of IFN-β in the respective tissues.

## RESULTS

### The anti-PRV activity of 25 flavonoids and primary screening for regulation of cGAS/STING signaling pathway were tested

**Fig 1:**
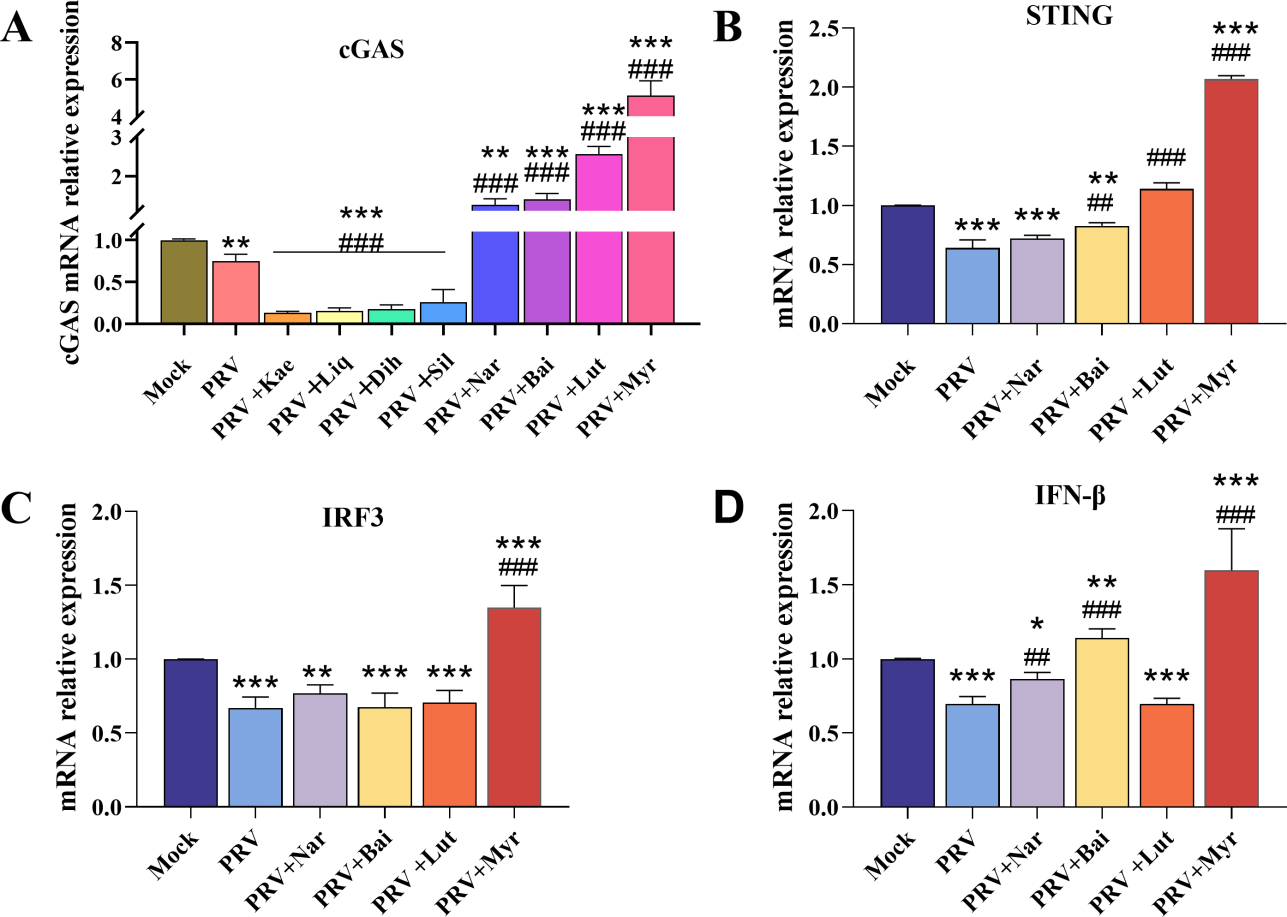
Effects of eight flavonoids on transcriptional levels of *cGAS*, *STING*, *IRF3,* and *IFN-β* in PRV-infected cells. Kae, kaempferol; Liq, liquiritigenin; Dih, dihydromyricetin; Sil, silymarin; Nar, naringenin; Bai, baicalein; Lut, luteolin; Myr, Myricetin. *, **, *** indicate *P* < 0.05, *P* < 0.01, and *P* < 0.001, respectively, when compared with the mock group. ## and ### indicate *P* < 0.01 and *P* < 0.001, respectively, when compared with the PRV group.

The study of anti-PRV activity revealed that eight flavonoids (resveratrol, baicalin, silymarin, myricetin, luteolin, dihydromyricetin, naringenin, and glycyrrhizic acid) exhibited notable antiviral activity, with IC_50_ values ranging from 23.24 to 323.09 µM. Hence, myricetin exhibited the highest activity (Table 2). Then, the regulation effects of the eight-active compound on cGAS/STING signaling pathway were tested. As illustrated in Fig. 1A, in comparison to uninfected cells, the mRNA expression level of *cGAS* in the PRV-infected group was significantly reduced. Treatment with naringin, baicalin, luteolin, and myricetin could significantly enhance the *cGAS* level, even more than that of control group. The transcriptional levels of key genes of the cGAS/STING signaling pathway, including *STING* (Fig. 1B), *IRF3* (Fig. 1C), and *IFN-β* (Fig. 1D), were also inhibited by PRV infection. The baicalin, luteolin, and myricetin could significantly increase in the mRNA level of *STING*. There were no discernible differences in the *IRF3* levels of naringin, baicalin, and luteolin when compared with the infected-control group. Only myricetin treatment could significantly increase the mRNA level of *IRF3*, even more than that of the control group. The naringenin, baicalin, and myricetin could significantly elevate the mRNA levels of *IFN-β* in comparison to the infected-control group, and the mRNA levels of *IFN-β* in the myricetin groups were higher than uninfected-control. These results suggested that among these compounds, myricetin could significantly activate the cGAS/STING signaling pathway that was inhibited by PRV infection. Therefore, myricetin was selected for further validation in the following tests

**TABLE 2:** Antiviral activity of 25 flavonoids against PRV

Serial number	Compound	CC50 (μM)	IC50 (μM)	SI^*a*^
1	Kaempferol	254.97	25.57	9.97
2	Baicalein	562.53	291.47	1.93
3	Fisetin	716.98	–^*b*^	–^*c*^
4	Diosmetin	606.14	–	–
5	Quercetin	890.08	–	–
6	Silibinin	392.29	203.26	1.93
7	Myricetin	>1000	42.69	23.24
8	Rutin	>389	–	–
9	Scutellarin	>212.93	–	–
10	Polydatin	>466.2	–	–
11	Quercetin	449.07	–	–
12	Chrysin	106.59	–	–
13	Lutelin	195.2	36.87	5.29
14	Dihydromyricetin	914.32	161.34	5.68
15	Palmatine	1251.8	–	–
16	Formononetin	228.46	–	–
17	Neohesperidin	>1000	–	–
18	Hesperidin	>237.9	–	–
19	Icariin	>382	–	–
20	Naringenin	>427.5	269.43	1.59
21	Mangiferin	>1050	–	–
22	Naringenin	1257.01	–	–
23	Liquiritigenin	790.04	323.09	2.45
24	Pueraria	>461.1	–	–
25	Biochanin A	381.56	–	–

^*a*^ SI = CC_50_/IC_50_.

^*b*^ In the IC50 column, a dash (–) indicates that the drug in question has no effect against PRV.

^*c*^ In the SI column, a dash (–) signifies that the specific value could not be calculated.

### The regulation of the cGAS/STING signaling pathway by myricetin following PRV infection was conducted

**Fig 2:**
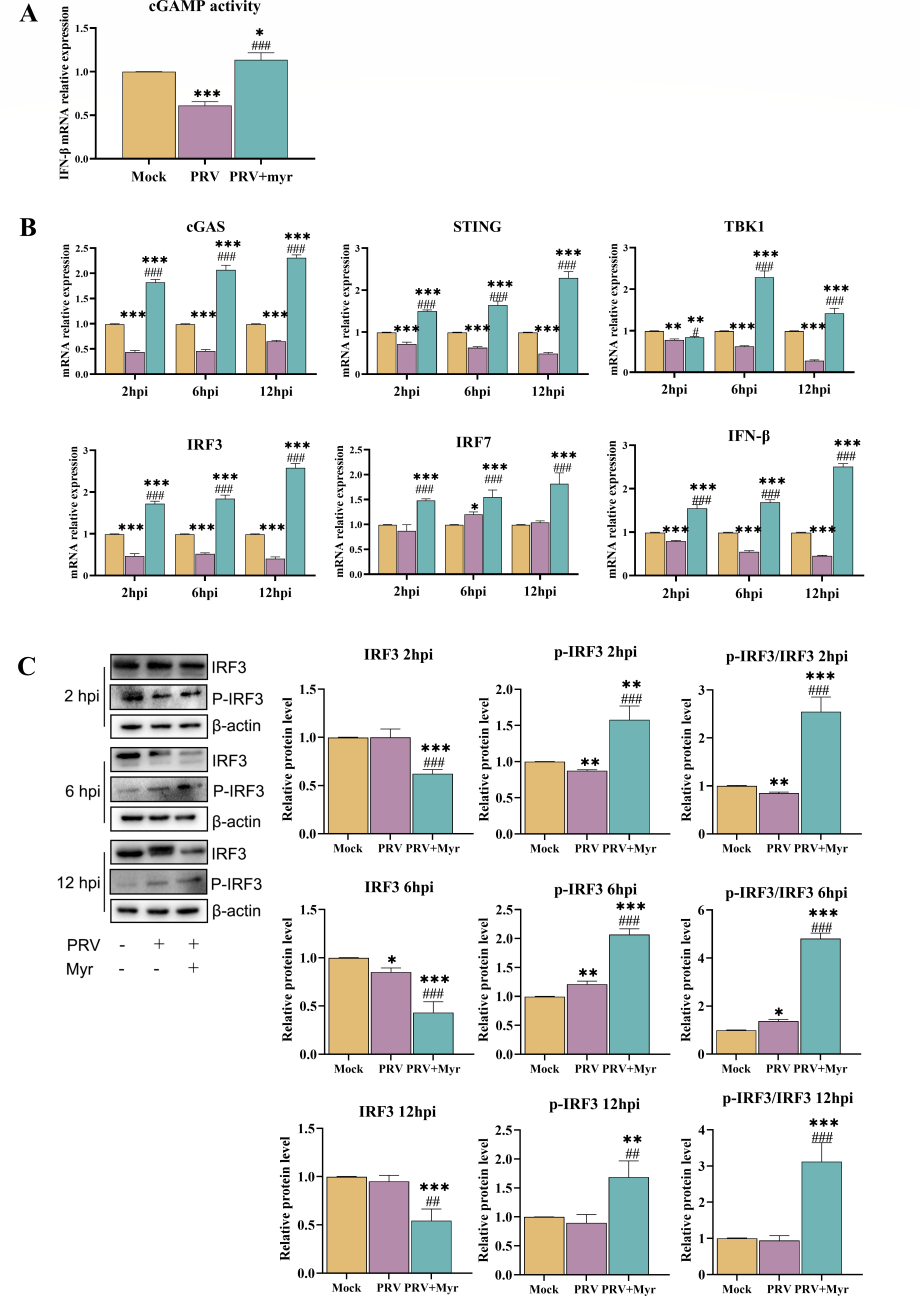
The regulation of the cGAS/STING signaling pathway by myricetin in PRV-infected cells. (**A**) Effect of myricetin on cGAMP production after PRV infection. (**B**) Effects of myricetin on transcriptional levels of *cGAS*, *STING*, *IRF3*, *IRF7*, *TBK1,* and *IFN-β* at 2, 6, and 12 hpi. (**C**) The expressions of IRF3 and p-IRF3 proteins in cells at 2, 6, and 12 hpi. Mock, uninfected-untreated group; PRV, infected-untreated group; PRV + Myr, infected group treated with myricetin. *, **, *** indicate *P* < 0.05, *P* < 0.01, and *P* < 0.001, respectively, when compared with the mock group. #, ##, and ### indicate *P* < 0.01 and *P* < 0.001, respectively, when compared with the PRV group.

During the activation of the cGAS/STING signaling pathway, cyclic guanosine monophosphate (cGAMP), an important second messenger of the pathway, is synthesized in large quantities (30). Consequently, cGAMP was extracted from the cells of each treatment group and subsequently added to normal cells. The alterations in cGAMP production were then indirectly reflected by the detection of the mRNA expression level of *IFN-β* in the cells. As illustrated in Fig. 2A, the production of *cGAMP* in the viral group was found to be significantly lower than that observed in the control group. In contrast, myricetin treatment significantly enhanced the synthesis of *cGAMP*, resulting in a nearly 1.5-fold increase in *cGAMP* production compared with the blank group. These results suggested that myricetin treatment was able to promote the signal transduction of the cGAS/STING signaling pathway after PRV infection.

At 2, 6, and 12 h post-infection (hpi), the transcriptional levels of key genes of cGAS/STING signaling pathway, including *cGAS*, *STING*, *TBK1*, *IRF3*, *IRF7,* and *IFN-β* were evaluated (Fig. 2B). The transcriptional levels of these genes were all inhibited by PRV infection when compared with uninfected group. After treatment with myricetin, the mRNA levels of these genes were all increased in comparison to the infected control and higher than those of the uninfected control.

**Fig 3:**
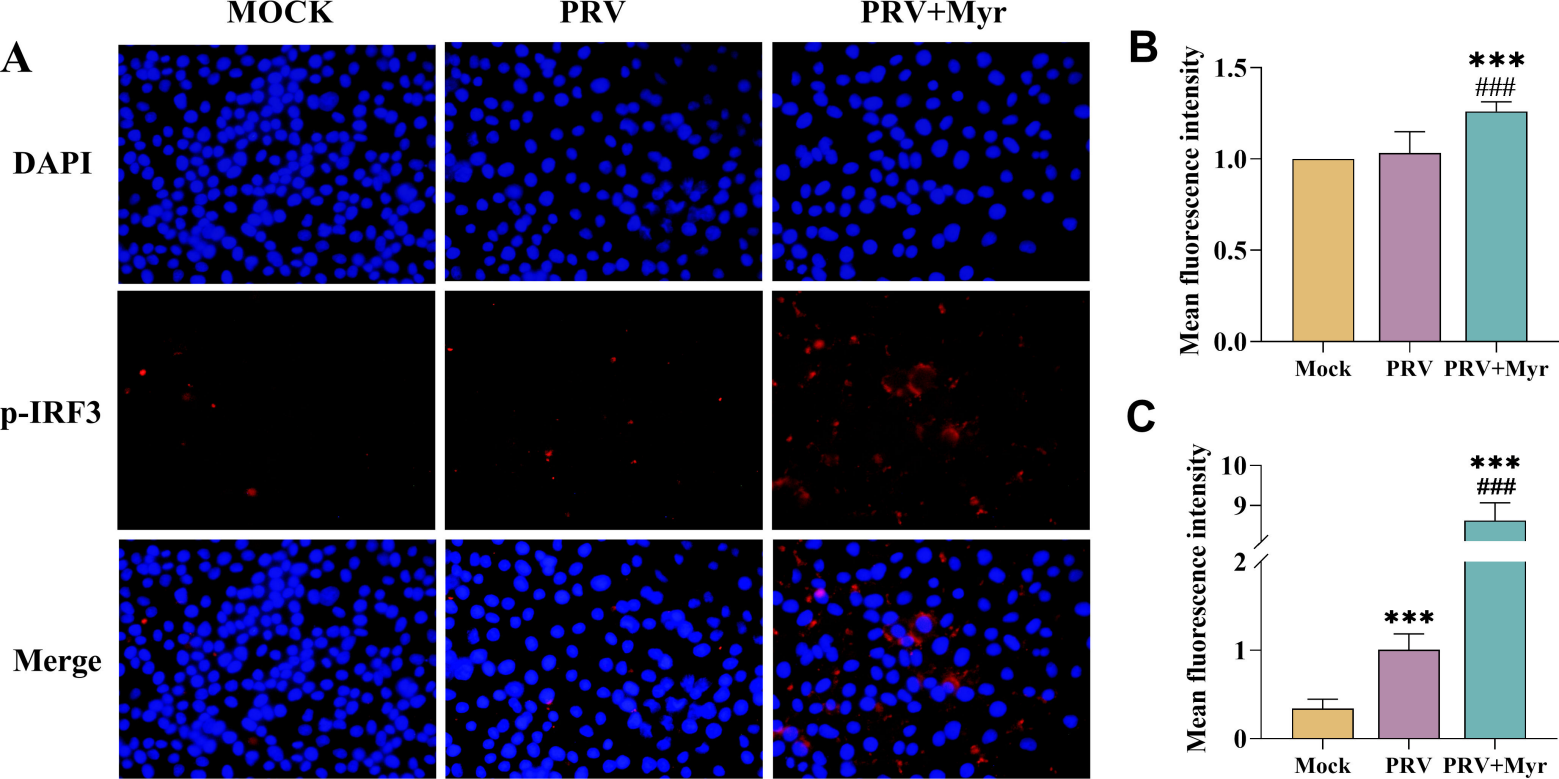
Effect of myricetin on the expression of phosphorylated IRF3 by indirect immunofluorescent assay. (**A**) The representative images of indirect immunofluorescent. (**B**) The fluorescence intensity in the nucleus. (**C**) The total fluorescence intensity. Mock, uninfected-untreated group; PRV, infected-untreated group; PRV + Myr, infected group treated with myricetin. *** indicates *P* < 0.001 when compared with the mock group. ### indicates *P* < 0.001 when compared with the PRV group.

Upon activation of the cGAS/STING signaling pathway, IRF3 undergoes phosphorylation and dimerization, after which it enters the nucleus and activates the production of type I interferon and downstream interferon-stimulated genes. Therefore, the contents of IRF3 and phosphorylated IRF3 were determined at 2, 6, and 12 hpi (Fig. 2C). A significant reduction in the p-IRF3/IRF3 ratio was observed in PRV-infected group at 2 hpi, which then increased at 6 hpi and subsequently returned to a level comparable with that of the uninfected control at 12 hpi. The levels of p-IRF3 and p-IRF3/IRF3 were significantly elevated in the myricetin-treated group at 2, 6, and 12 hpi, in comparison to the infected-control and uninfected-control groups. The overall activation status of the pathway was also determined by observing the expression of phosphorylated IRF3 in the nucleus by indirect immunofluorescence (Fig. 3). The total amount of IRF3 phosphorylation was observed to be elevated in the PRV-infected group in comparison to the mock group (Fig. 3C). Myricetin treatment resulted in a significant enhancement of the total amount of IRF3 phosphorylation in comparison to the PRV-infected group, as well as an increase in the amount present in the nucleus (Fig. 3B).

The results indicated that at the early stage of PRV infection (2 hpi), the cGAS/STING signaling pathway was inhibited. With viral multiplication, the pathway was activated at 6 hpi and gradually returned to a normal level at 12 hpi. Myricetin could maintain a high level of activation of the cGAS/STING signaling pathway during PRV infection.

### The regulation of the JAK/STAT signaling pathway by myricetin following PRV infection was conducted

**Fig 4:**
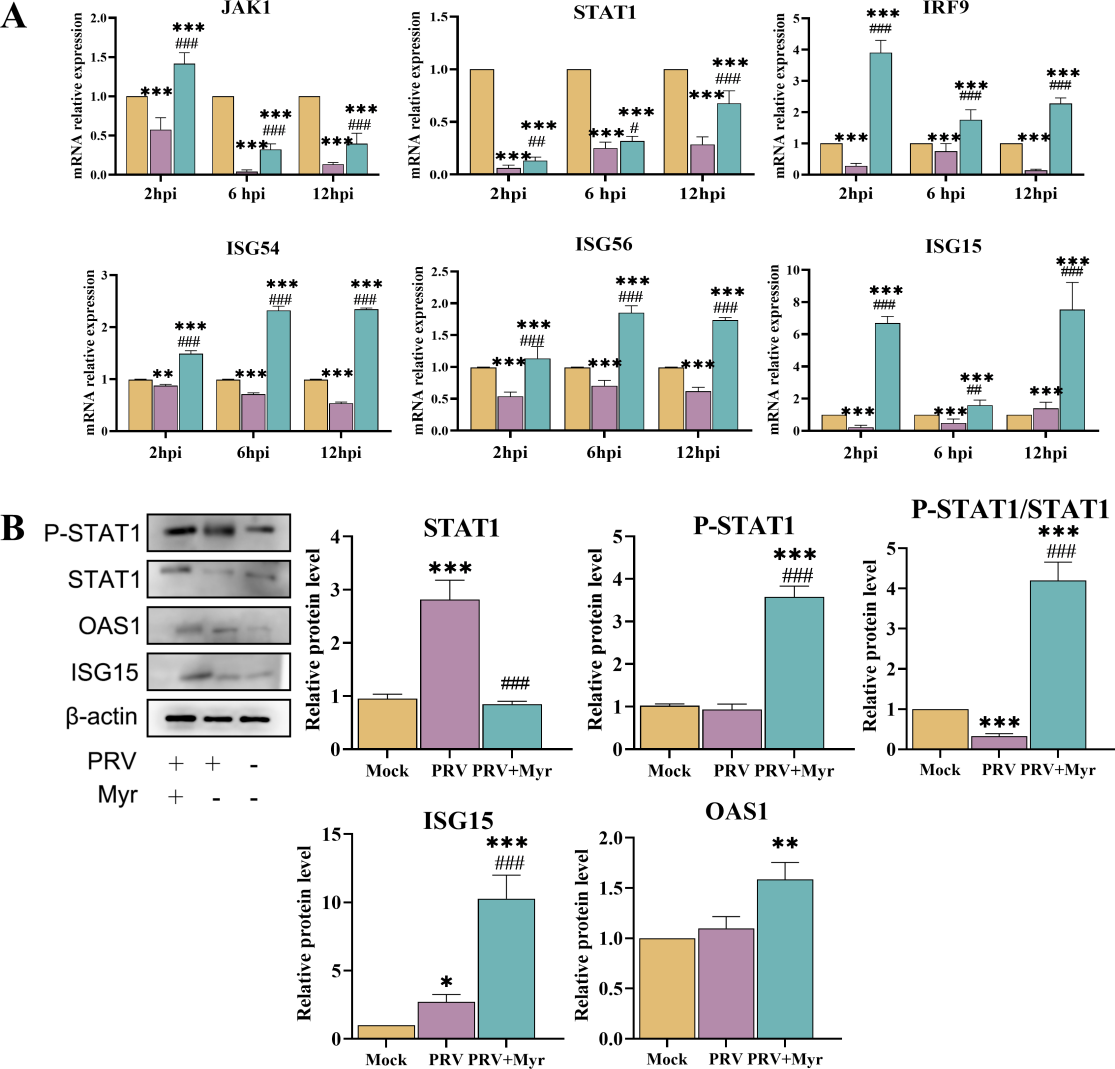
The regulation of the JAK/STAT signaling pathway by myricetin in PRV-infected cells. (**A**) Effects of myricetin on transcriptional levels of JAK1, STAT1, ISG15, ISG54, and ISG56. (**B**) The expressions of STAT1, P-STAT1, ISG15, and OAS1 proteins. Mock, uninfected-untreated group; PRV, infected-untreated group; PRV + Myr, infected group treated with myricetin. *, **, *** indicate *P* < 0.05, *P* < 0.01, and *P* < 0.001, respectively, when compared with the mock group. #, ##, and ### indicate *P* < 0.01 and *P* < 0.001, respectively, when compared with the PRV group.

Upon the activation of the cGAS pathway, IFN is secreted outside the cell and binds to the receptors (IFNAR1 and IFNAR2) on the cell membrane, thereby activating the JAK/STAT signaling pathway. This process generates the antiviral effector proteins, ISGs, which are responsible for the antiviral effects (31). The transcriptional levels of key genes of the JAK-STAT pathway, including *JAK1*, *STAT1*, *IRF9*, *ISG15*, *ISG56*, and *ISG54*, were quantified and found to be inhibited by PRV infection in comparison to the uninfected control group (Fig. 4A). Treatment with myricetin resulted in a notable elevation in the transcriptional levels of these genes, with levels significantly higher than those observed in the uninfected control group.

The expression levels of key proteins of the JAK-STAT pathway, including STAT1, p-STAT1, ISG15, and OAS1, are presented in Fig. 4B. There was no significant difference in the protein expression of STAT1 and p-STAT1 in the viral group compared with uninfected control group, suggesting that PRV infection inhibited the activation of the JAK-STAT pathway. In contrast, the protein levels of STAT1, p-STAT1, and p-STAT1/STAT1 were found to be significantly higher in myricetin-treated group, suggesting that myricetin could activate the pathway during PRV infection. These changes were also demonstrated by the results of effect proteins ISG15 and OAS1, which was significantly increased in myricetin-treated group during PRV infection.

### The activation of the type I interferon signaling pathway by myricetin following PRV infection has been confirmed in HEK-293 cells

**Fig 5:**
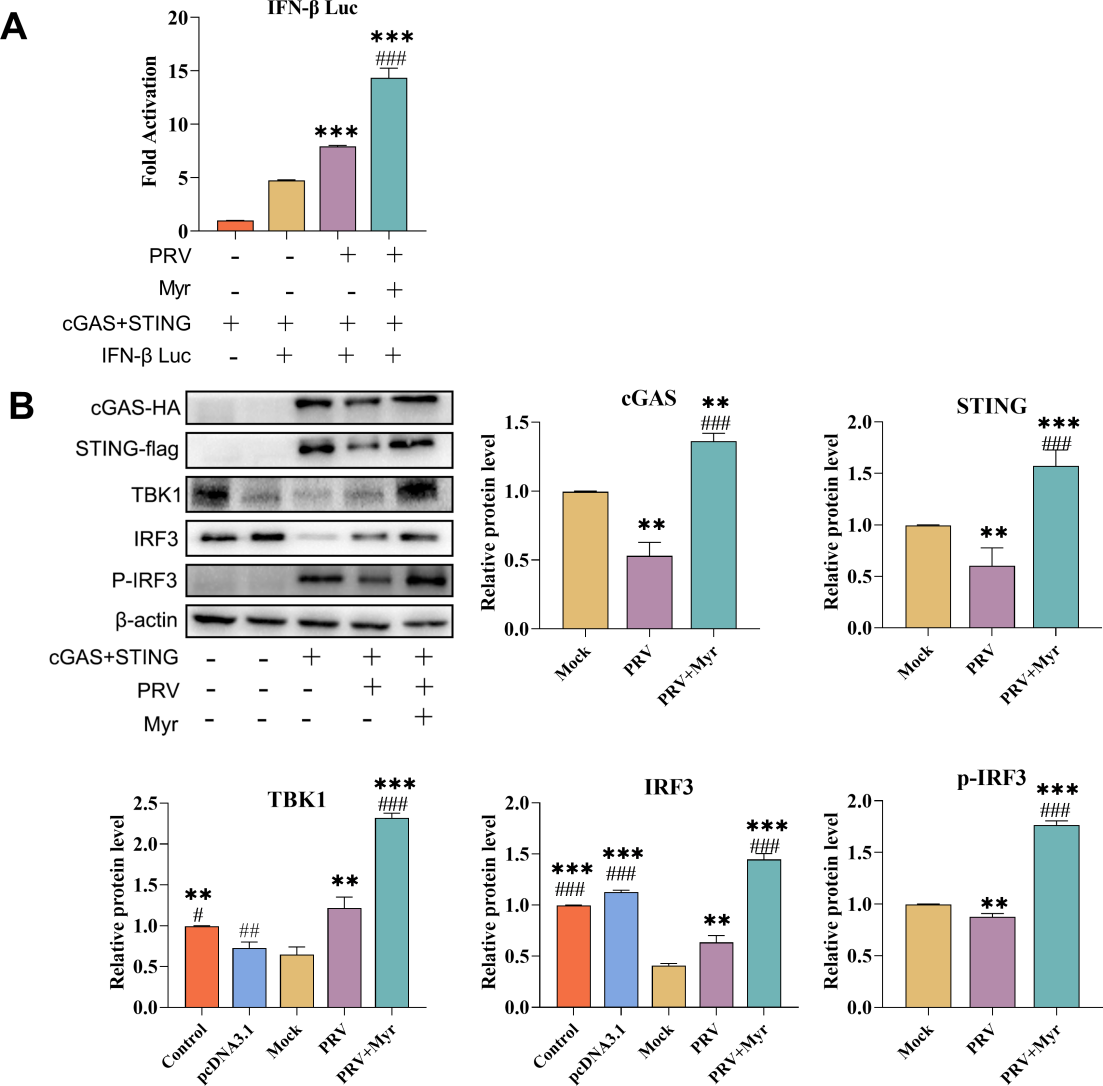
The regulatory effects of myricetin on the type I interferon signaling pathway in HEK-293 cells. (**A**) Evaluation of IFN-β promoter expression by a dual luciferase gene reporter assay. (**B**) The protein expression of cGAS/STING signaling pathway. Control, the HEK-293 cells without any treatment; pcDNA3.1, the cells transfected with pcDNA3.1 control plasmid; Mock, the cells were transfected with cGAS-HA and STING-flag plasmids; PRV, the cells were transfected with cGAS-HA and STING-flag plasmids and infected with PRV; PRV + Myr, the cells were transfected with cGAS-HA and STING-flag plasmids and infected with PRV in the presence of myricetin. *, **, *** indicate *P* < 0.05, *P* < 0.01, and *P* < 0.001, respectively, when compared with the mock group. #, ##, and ### indicate *P* < 0.01 and *P* < 0.001, respectively, when compared with the PRV group.

To confirm the activation of the type I interferon signaling pathway by myricetin, HEK-293 cells were transfected with cGAS-HA and STING-flag plasmids to establish a cellular model of the cGAS/STING signaling pathway. Subsequently, a dual luciferase gene reporter assay was conducted to assess the impact of myricetin on the expression of *IFN-β* during PRV infection in this model. As illustrated in Fig. 5A, the expression of the IFN-β promoter was found to be elevated following the transfection of the cGAS and STING plasmid. In comparison to the uninfected control group, the expression of the *IFN-β* promoter was observed to be elevated in the PRV-infected group. However, the expression level of the *IFN-β* promoter in the myricetin-treated group was found to be significantly higher than that in the infected control group, with an approximate 2-fold ratio. This indicates that myricetin enhances the expression of the pathway effector IFN-β following PRV infection.

The protein expression levels of cGAS-HA, STING-flag, TBK1, IRF3, and p-IRF3 in the transfected HEK-293T cells were detected to validate the activation of the pathway by myricetin. As illustrated in Fig. 5B, the cGAS, STING, and p-IRF3 proteins were not expressed in the blank control and plasmid control groups. After transfection, these proteins could be all detected. In the transfected-infected group, the protein expressions of cGAS, STING, and p-IRF3 were significantly inhibited in comparison with the transfected control group. In contrast, myricetin could significantly increase the expressions of cGAS, STING, TBK1, IRF3, and p-IRF3 when compared with the transfected-uninfected and transfected-infected groups. These results indicated that myricetin could activate the cGAS/STING signaling pathway, which was inhibited by PRV infection.

### The activation of the type I interferon signaling pathway by myricetin has been confirmed in PRV-infected mice

A PRV-infected mouse model was established for further validation in accordance with the previously described methods (32, 33). As previously demonstrated (32), the mortality rate in the infected-untreated group reaches 40% at 4 days post-infection (dpi), subsequently increasing to 70% at 5 dpi. In the group treated with myricetin, the mortality rate was 0% at 4 dpi and then increased to 30% at 5 dpi. The viral load in the kidney, liver, lung, spleen, and brain was quantified by determining the number of genomic copies of PRV. Myricetin treatment resulted in a significant inhibition of viral proliferation in the test organs, with a reduction in viral copies of up to 80-fold in the kidney.

**Fig 6:**
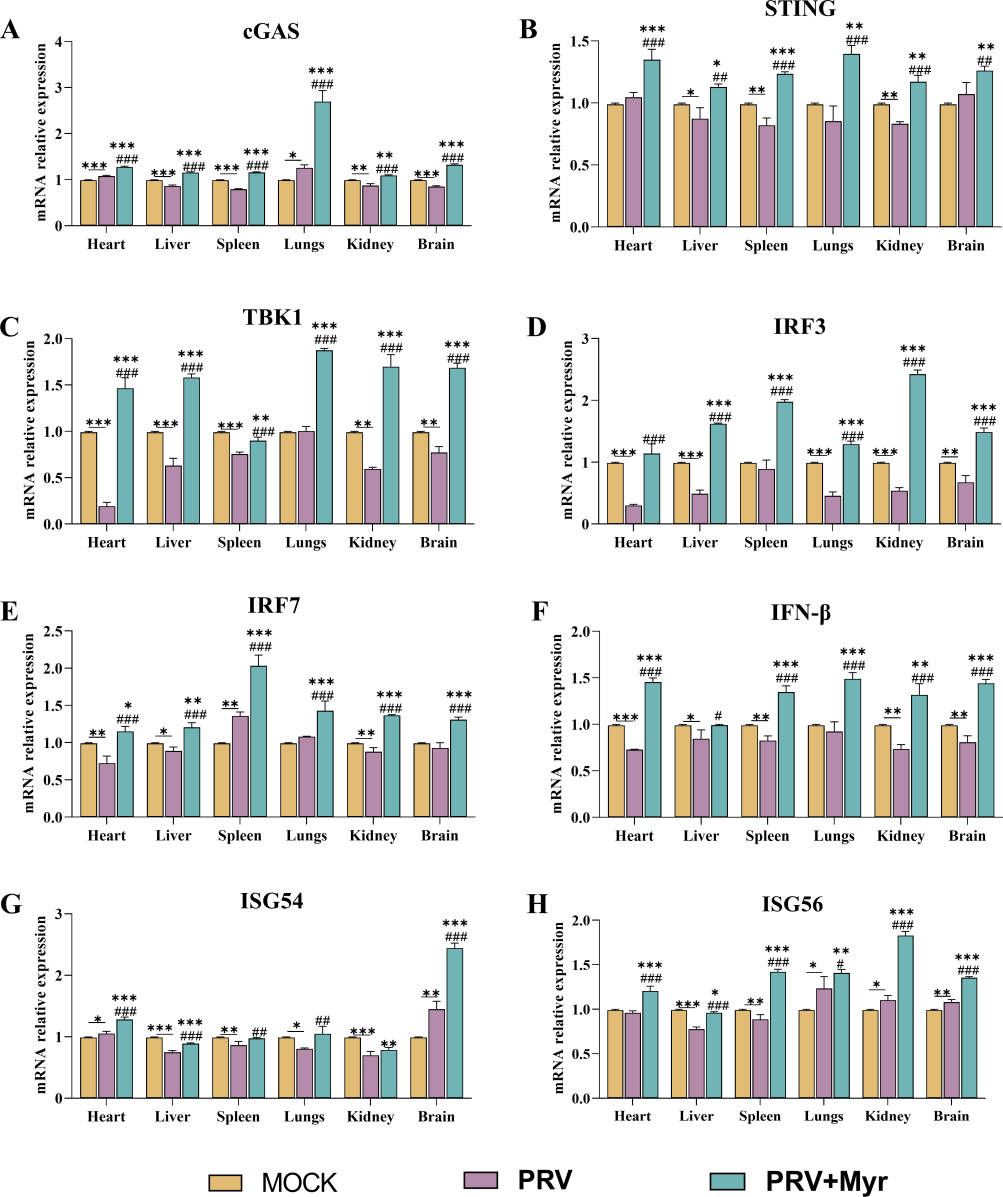
The transcriptional levels of cGAS, STING, TBK1, IRF3, IRF7, ISG54, ISG56, and IFN-β in mice. Mock, uninfected-untreated group; PRV, infected-untreated group; PRV + Myr, infected group treated with myricetin. *, **, *** indicate *P* < 0.05, *P* < 0.01, and *P* < 0.001, respectively, when compared with the mock group. #, ##, and ### indicate *P* < 0.01 and *P* < 0.001, respectively, when compared with the PRV group.

The transcriptional levels of key genes involved in the type I interferon signaling pathway in the the heart, liver, spleen, lung and kidney, including *cGAS*, *STING*, *TBK1*, *IRF3*, *IRF7*, *IFN-β*, *ISG56,* and *ISG54*, were evaluated (Fig. 6). The results indicated a general downward trend or slight upward trend in the levels of these genes in PRV-infected mice when compared with uninfected mice, especially the levels of *IFN-β* were decreased in all test organs. In the myricetin-treated groups, the levels of the tested genes were all significantly higher than those observed in the uninfected and infected control groups.

**Fig 7:**
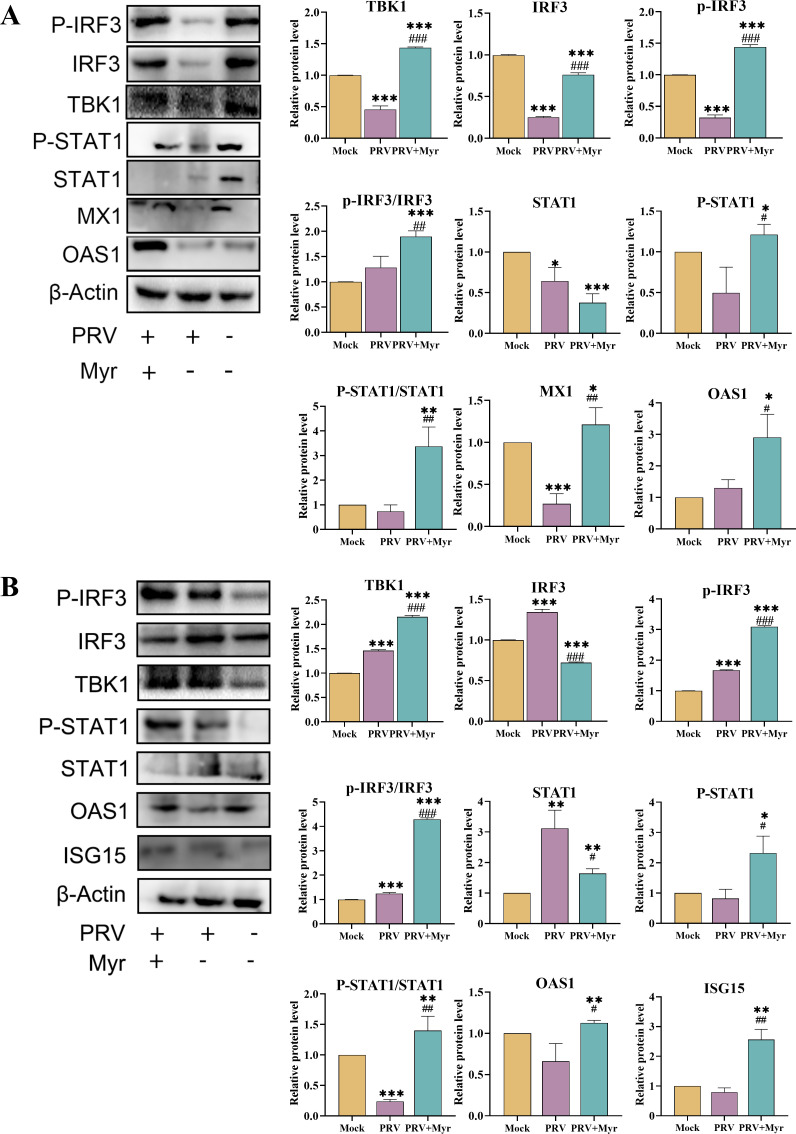
The protein expressions of the type I interferon signaling pathway in the brain (**A**) and kidney (**B**) of mice. Mock, uninfected-untreated group; PRV, infected-untreated group; PRV + Myr, infected group treated with myricetin. *, **, *** indicate *P* < 0.05, *P* < 0.01, and *P* < 0.001, respectively, when compared with the mock group. #, ##, and ### indicate *P* < 0.01 and *P* < 0.001, respectively, when compared with the PRV group.

**Fig 8:**
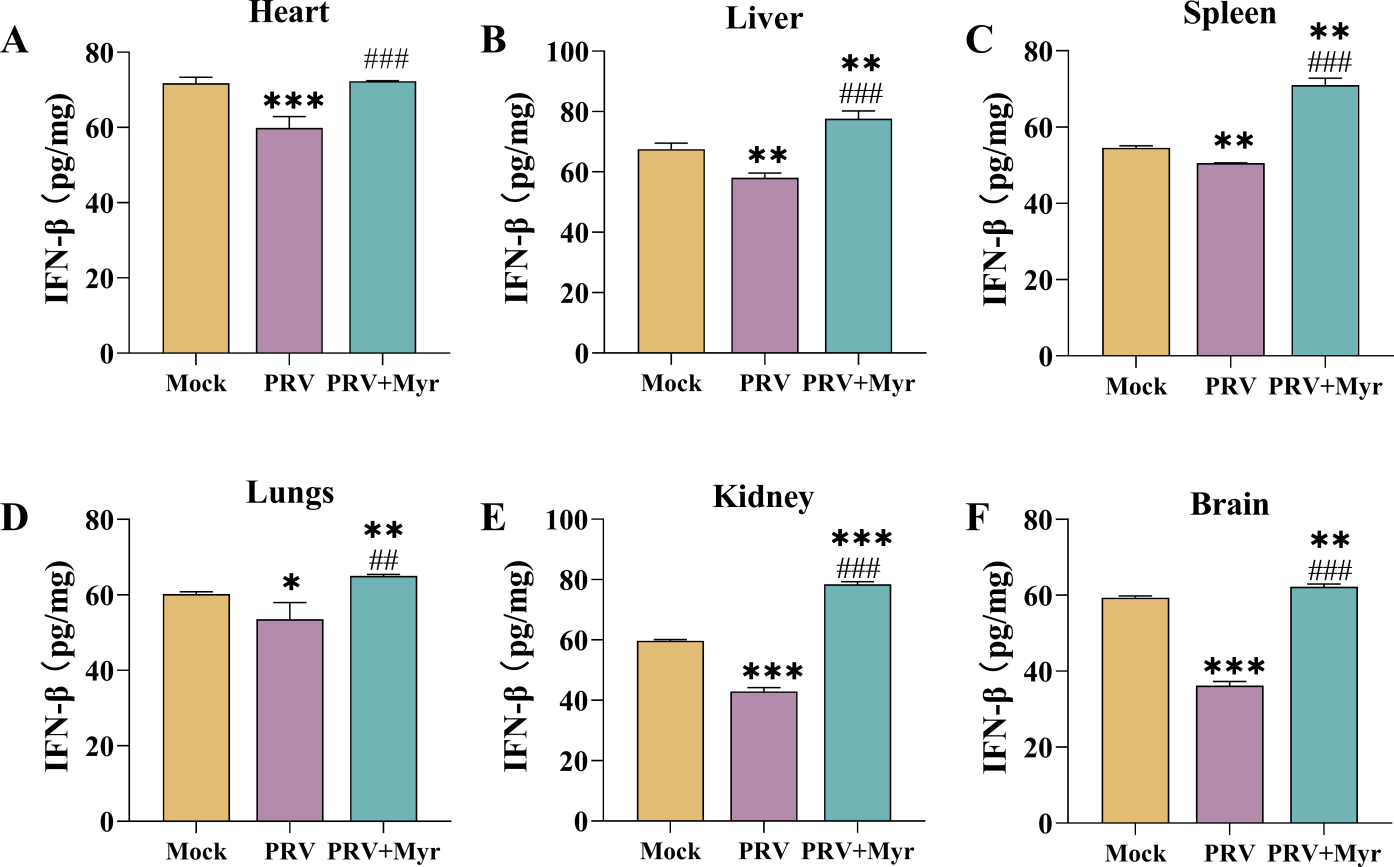
IFN-β contents in the heart, liver, spleen, lung, kidney, and brain of mice. Mock, uninfected-untreated group; PRV, infected-untreated group; PRV + Myr, infected group treated with myricetin. *, **, *** indicate *P* < 0.05, *P* < 0.01, and *P* < 0.001, respectively, when compared with the mock group. #, ##, and ### indicate *P* < 0.01 and *P* < 0.001, respectively, when compared with the PRV group.

The brain and kidney were the primary target organs of PRV (33), and thus, the protein expressions of TBK1, IRF3, p-IRF3, STAT1, P-STAT1, OAS1, MX1, and ISG15 in the two organs were evaluated (Fig. 7). PRV infections did not cause the activation of cGAS/STING and JAK/STAT pathways in the brain due to the unchanged levels of OAS1, p-IRF3/IRF3, and P-STAT1/STAT1, and the expression of MX1 was decreased. In the kidney, PRV infections increased the levels of p-IRF3/IRF3, but decreased the levels of P-STAT1/STAT1; the expressions of antiviral effectors OAS1 and ISG15 did not show any changes in comparison with uninfected control. Treated with myricetin activated these pathways and induced higher expression levels of antiviral effectors MX1, OAS1, and ISG15. These results suggested that PRV has the capacity to evade the body’s immune response, thereby inhibiting the synthesis of IFN-β (34). The contents of IFN-β in the heart, liver, spleen, lung, and kidney were all significantly decreased after PRV infection, and myricetin could significantly promote the production of IFN-β in mice (Fig. 8), thereby enhancing the body’s antiviral capacity.

## DISCUSSION

Flavonoids, which are natural substances widely found in plants, are widely known for their biological activities and have been shown to have unique activities in the treatment of a variety of viral infections, including herpesviruses (35, 36). In this study, 25 flavonoids with reported antiviral activities were selected for *in vitro* screening of anti-PRV activity. Considering the IC50 values (37), eight compounds with potent antiviral activity against PRV were identified, namely kaempferol, baicalein, silymarin, myricetin, lignocerin, dihydropyronin, naringenin, and glycyrrhizin. These compounds can be considered candidates for the development of anti-PRV drugs from natural products.

The cGAS/STING signaling pathway plays a pivotal role in the course of herpesvirus infection (38), serving as the primary signaling pathway that recognizes dsDNA signals within the cell and inducing the onset of the major type I interferon response (39). Nevertheless, it has been demonstrated that PRV infection exerts an inhibitory effect on the cGAS/STING signaling pathway through viral UL13 protein, which can promote the degradation of STING proteins. The gI protein, in turn, interferes with the formation of dimers from phosphorylated IRF3, thereby disrupting the signaling pathway. The expression of the gE protein can inhibit the secretion of the pathway effector IFN-β (40). To investigate whether the eight active flavonoids could activate the cGAS/STING signaling pathway after PRV infection, the gene expression of cGAS, a pathway sensing factor, was initially examined in the early stage of PRV infection. It was found that PRV reduced the expression of cGAS, indicating that PRV could inhibit the activation of the pathway. However, the expression level of the cGAS gene was found to be significantly up-regulated following treatment with the four compounds, naringnin, baicalein, lignanin, and myricetin. Further analysis of the expression of downstream genes revealed that myricetin was the only compound to promote the expression of STING, IRF3, and IFN-β in the case of inhibition by PRV infection. The results demonstrate that myricetin exerts anti-PRV activity through the upregulation of the activation level of the cGAS/STING signaling pathway following PRV infection. Although kaempferol exhibited the highest antiviral efficacy against PRV, our previous research has indicated that its antiviral mechanism does not involve the IFN-I pathway. This is primarily due to the inhibition of the transcriptional levels of the immediate early gene IE180 and the early genes (EPO and TK) (41). These results were consistent with those of the present study.

The cGAS/STING signaling pathway is present in most animal cells, where cGAS acts as the DNA receptor that enables signaling in this pathway and as a nucleotidyltransferase that promotes the synthesis of the second messenger cGAMP (41, 42). Conversely, viral infections can evade cGAS in several ways, including recognition. Porcine circovirus type 2 has been demonstrated to induce phosphorylation of the cGAS protein at S278 through the PI3K/Akt signaling pathway, which directly inhibits its catalytic activity (43). In contrast, during PRV infection, the UL21 protein of the virus degrades cGAS through autophagy reactions mediated by the shipping receptor Toll-IP. This process evades immune recognition and renders cGAS incapable of receiving exogenous dsDNA signaling stimuli (44, 45). The present study examined the mRNA expression levels of cGAS and the content of cGAMP, a molecule downstream of the cGAS pathway, as reflected by the mRNA expression levels of IFN-β (46). The results indicated that myricetin could release the inhibitory effect of PRV on cGAS gene expression and cGAMP production. This resulted in an increase in the expression level of the cGAS gene and an improvement in cGAMP production, which promoted the recognition of intracellular viral dsDNA by cGAS and the normal synthesis of the second messenger cGAMP.

This study demonstrated that PRV infection could reduce the mRNA expression level of the STING gene. However, treatment with myricetin significantly enhanced the expression of the STING gene. This suggests that myricetin may limit the degrading effect of PRV-associated viral proteins on STING, thereby enabling the normal conduction of pathway signals. However, STING can promote the phosphorylation and dimerization of IRF3 under the action of TBK1 and indirectly promote the phosphorylation of IRF3 and IRF7, which can then enter the nucleus and activate the production of downstream interferon-stimulated genes (47). The findings of this study indicate that PRV infection resulted in a notable inhibitory impact on the mRNA expressions of pathway-conducting genes (TBK1, IRF3, and IRF7). However, this inhibitory effect was reversed by myricetin. Additionally, it elevated the mRNA expression levels of the downstream type I interferon response effector IFN-β, its mediated JAK-STAT pathway, and interferon-stimulated genes (ISG54, ISG56, and ISG15). This suggests that myricetin can overcome the inhibition of the cGAS/STING signaling pathway by PRV via the “TBK1-IRF3” pathway (48).

The cGAS/STING pathway, which transmits signals from the cytoplasm to the nucleus to initiate the type I interferon response, is mediated by IRF3 phosphorylation and dimerization, which shifts into the nucleus (49). Conversely, viral infections can impede the phosphorylation, dimerization, or nucleation processes of IRF3, thereby impeding the normal transmission of signals (29, 50). The VP24 protein of HSV-1 reduces the transcription initiation of the IRF3 gene and restricts its phosphorylation and dimerization process (51). Conversely, following infection of host cells with PRV, the UL13 protein can inhibit the phosphorylation of IRF3, thereby preventing the transmission of signals to the nucleus (52, 53). Therefore, we examined the level of phosphorylated IRF3 in the nucleus and the expressions of IRF3 and p-IRF3 proteins. Our findings demonstrated that myricetin could promote the phosphorylation of IRF3 into the nucleus and increase the protein expression level of p-IRF3. Consequently, it can be concluded that myricetin can counteract the inhibitory effect of PRV on the cGAS/STING signaling pathway.

The cGAS and STING molecules can exert anti-DNA viral infection in host cells by recognizing DNA molecules and regulating the expression of host interferon (IFN-β) and pro-inflammatory cytokines via a cascade amplification response (54). Some pathway agonists have been demonstrated to have a stimulatory effect on the cGAS/STING signaling pathway, thereby inducing the occurrence of a type I interferon response in the organism (55). It has been demonstrated that cisplatin can enhance the recognition of chromatin in cancer cells by the cGAS protein, which in turn activates the signaling pathway and exerts an inhibitory effect on the proliferation of bladder cancer cells. Cyclic dinucleotide agonists, such as 2′5′-cGAMP, can enhance the activation level of STING proteins and activate the cGAS/STING signaling pathway in neighboring cells via transport vesicles as well as intercellular junctions, thereby improving immune recognition of viral particles or tumor cells (56, 57). HEK-293T cells, which are commonly used for exogenous gene overexpression, have high expression efficiency for the corresponding expressed proteins after eukaryotic plasmid transfection (58). However, the absence of cGAS and STING proteins in these cells precludes their use as a model for studying this DNA transduction signaling pathway. Therefore, overexpression of a cGAS and STING in HEK293T cells can effectively compensate for this deficiency and activate IFN-β expression after transfection (59). In the cell model, we confirmed that myricetin significantly increased the expression of the IFN-β promoter after PRV infection, suggesting that myricetin enhanced the occurrence of type I interferon response. The expressions of pathway-related proteins (cGAS, STING, TBK1, IRF3, and p-IRF3) in the cell model were also detected, which showed some trends observed in PK-15 cells. Combined with these results, we can see that myricetin can regulate the cGAS/STING signaling pathway inhibited by PRV, promote the expression of key proteins of the pathway, and then limit the occurrence of viral immune evasion.

To verify that myricetin can regulate the type I interferon response to inhibit PRV proliferation, as well as activate this pathway in animals to exert anti-PRV effects, a PRV-infected mouse model was also used (60). Myricetin can improve the manifestation symptoms of PRV infection in mice and extend their survival time (32). In this study, myricetin has been observed to increase the transcriptional expression level of pathway-related genes in heart, liver, spleen, lung, kidney, and brain tissues of mice. Additionally, it increased the protein expressions of TBK1 and p-IRF3 in brain and kidney tissues, which activates the cGAS/STING signaling pathway in mice. Furthermore, it also induced the secretion of IFN-β in tissues. This modulation enhances the ability of mice to resist PRV infection and limits the excessive immune response due to persistent viral infection (61), thereby reducing the organismal damage caused by an imbalance of the immune system (62). It can be demonstrated that myricetin can activate the cGAS/STING signaling pathway in mice, which was inhibited by PRV infection. This, in turn, exerts an inhibitory effect on PRV *in vivo* and *in vitro* by facilitating the onset of the type I interferon response.

**Fig 9:**
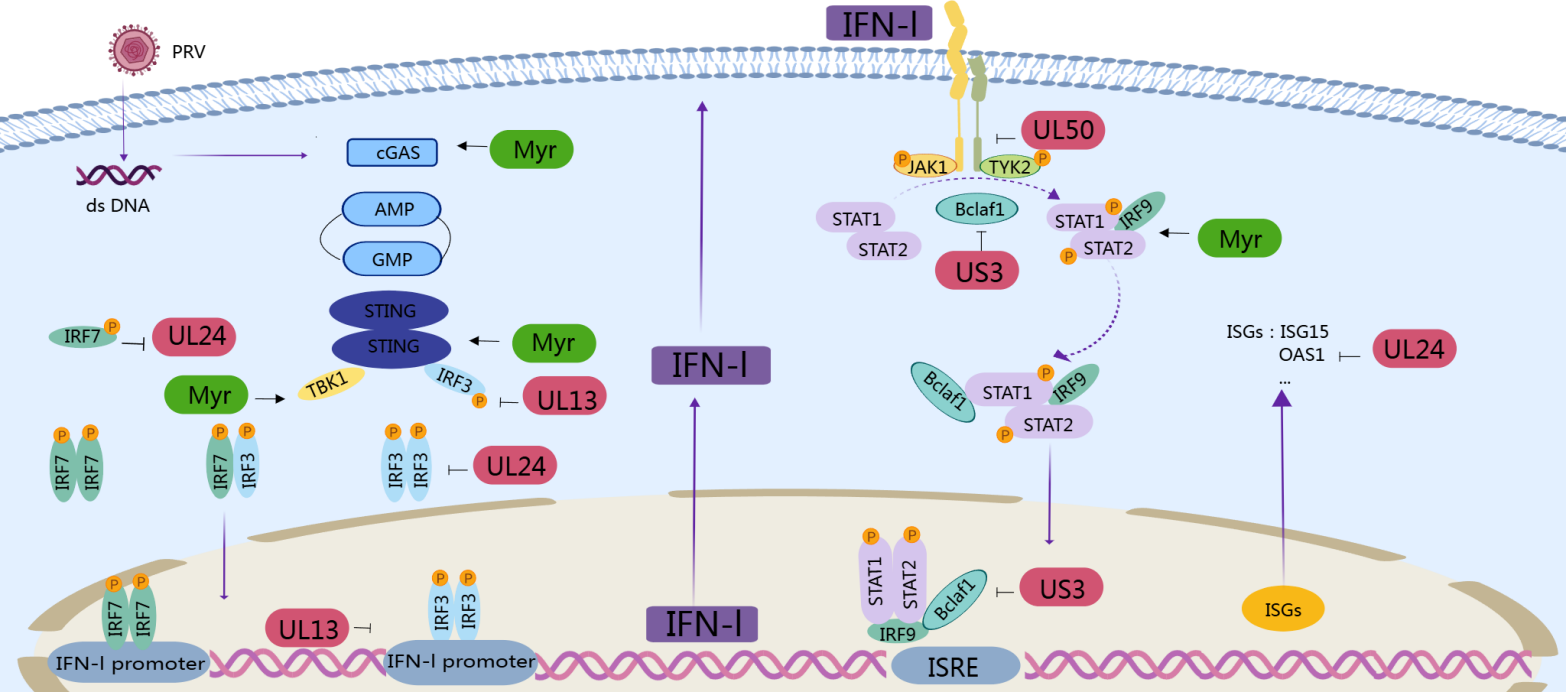
Schematic presentation of anti-PRV molecular mechanism of myricetin through the type I interferon signaling pathway.

The JAK/STAT signaling pathway is of paramount importance regarding the effect of interferon. The secreted IFN binds to IFN receptors 1 and 2 (IFNAR1 and IFNAR2) through autocrine and paracrine manners, thereby activating the downstream Janus kinases JAK1 and TYK2 (63). Subsequently, the relevant receptors are phosphorylated by the kinases, providing a docking platform for transcription factors STAT1 and STAT2 (64). The STAT proteins are then phosphorylated by JAK, forming ISGF3. ISGF3 then translocates to the nucleus and binds to ISRE, activating the transcription of a series of ISGs (65). The ISGs then synergistically inhibit viral replication within the host through various mechanisms. This process establishes an antiviral state that inhibits viral replication, stimulates the adaptive immune response, and recruits other immune cells to the site of infection (66, 67). The evasion of the JAK-STAT-mediated immune response by herpesviruses is achieved through the utilization of disparate mechanisms. The UL26USP protein of HSV-1 has been demonstrated to inhibit the expression of ISGs through competitively binding to IFNAR2, thereby blocking the binding of JAK1 to IFNAR2 (68). Additionally, the ICP27 protein has been observed to inhibit the phosphorylation of STAT1 (69). PRV has been demonstrated to inhibit the signal transduction of JAK1 and TY2 through proteases, thereby suppressing STAT1 phosphorylation induced by type I and III interferons and inhibiting interferon-induced ISG expression (70). PRV’s US3 has been demonstrated to inhibit the phosphorylation of STAT1 by degrading Bclaf1 (71). In the present study, PRV was capable of inhibiting STAT1 phosphorylation in both mice and PK-15 cells and reducing the mRNA expression levels of the JAK1 and STAT1 genes. Myricetin could promote the phosphorylation of STAT1, facilitating the generation of effector proteins (MX1, OAS1, and ISG15). This suggests that myricetin can inhibit the PRV-induced degrading effect of JAK1 and STAT1, thereby enabling the normal conduction of pathway signals. In a recent study, the C-terminal region of the PRV early protein EP0 domain can bind to the IRF9 promoter, thereby inhibiting the transcription and expression of IRF9 (72). This, in turn, leads to a reduction in the production of type I IFN and its downstream ISGs, which allows the virus to evade the host’s antiviral immune response. The findings of our study indicate that myricetin exerts a certain activating effect on IRF9 expression. It has been reported that the DNA polymerase-generating factor of PRV (UL42) can be activated in the nucleus through its DNA binding site by a trimeric complex formed with ISGF3 (73). A trimeric complex formed by P-STAT1, P-STAT2, and IRF9 competitively binds to the IFN-stimulated response element (ISRE) in the nucleus, thereby inhibiting the production of ISGs and leading to a weakening of the host’s antiviral status. The results in this study indicate that the expressions of ISG15, ISG54, and ISG56 were markedly elevated in the presence of myricetin. This indicates that myricetin could antagonize the immune escape of PRV through activation of the JAK/STAT signaling pathway.(Fig. 9)

## Data Availability

Data are available from the corresponding authors upon request.
